# Supplementary figures and images for: Development of a recombinase polymerase amplification assay with lateral flow dipstick (RPA-LFD) for rapid detection of Shigella spp. and enteroinvasive Escherichia coli
Source: PLoS One. 2022 Dec 12;17(12):e0278869. doi: 10.1371/journal.pone.0278869 (PMC9744308; doi:10.1371/journal.pone.0278869)

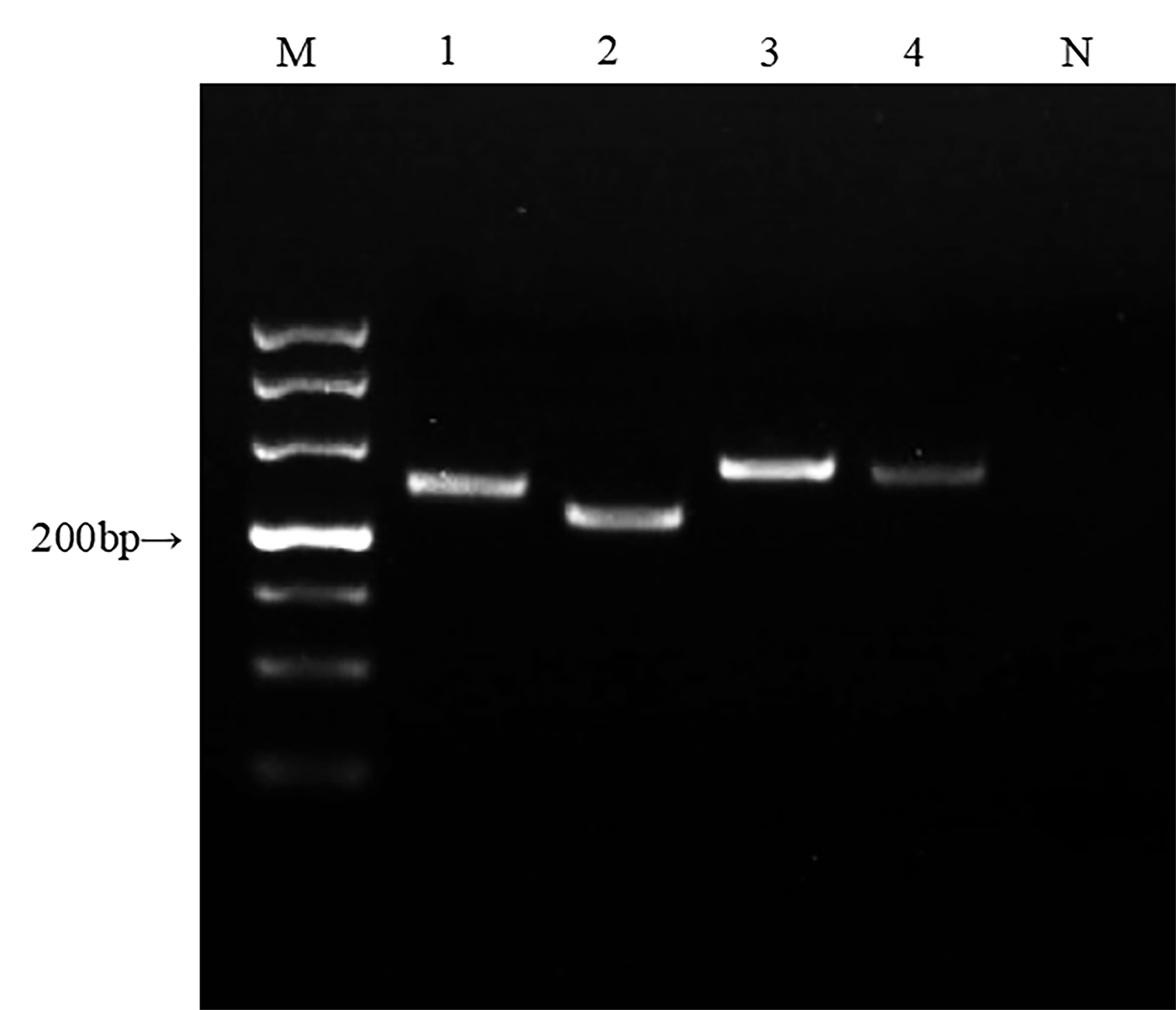

Supplement: S1 Fig — M: DNA marker. N: Negative control. 1–4: ipaH 01, ipaH 02, ipaH 03, ipaH 04. Under the same reaction conditions, the results of 3 agarose gel electrophoresis are more obvious. (TIF) [file pone.0278869.s001.tif]

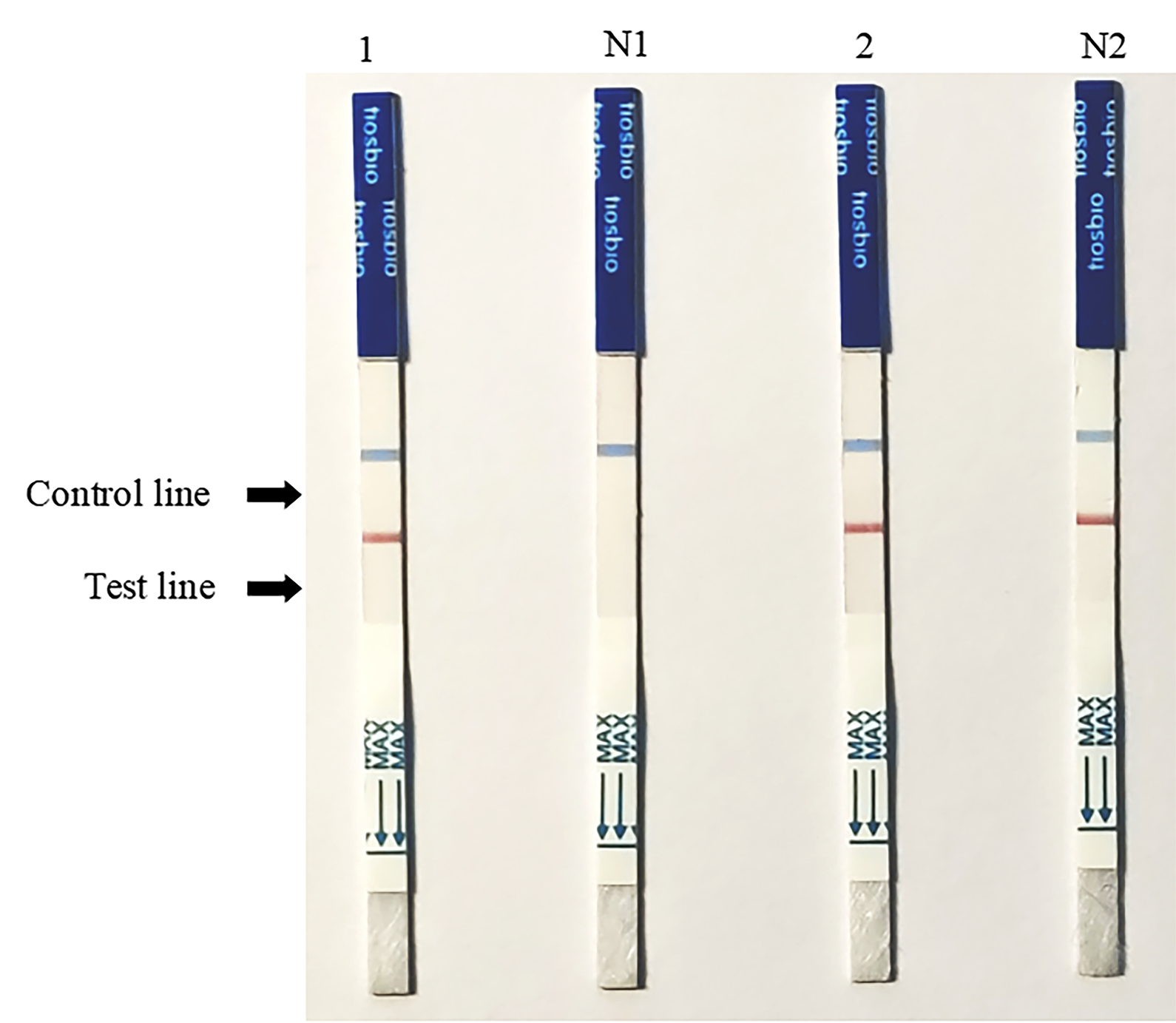

Supplement: S2 Fig — 1: ipaH probe1, 2: ipaH probe2, N1: ipaH probe1 negative control, N2: ipaH probe2 negative control. Probe 1 showed a positive result. In negative control 1, only the control line changed color, indicating it was a valid control. Probe 2 produced very obvious false positive results. (TIF) [file pone.0278869.s002.tif]
