# Supplementary material for: Development of a recombinase polymerase amplification assay with lateral flow dipstick (RPA-LFD) for rapid detection of Shigella spp. and enteroinvasive Escherichia coli
Source: PLoS One. 2022 Dec 12;17(12):e0278869. doi: 10.1371/journal.pone.0278869 (PMC9744308; doi:10.1371/journal.pone.0278869)
Supplement: S1 Table — (DOCX) [file pone.0278869.s003.docx]

**S1 Table**

| Primer name | Primer direction | Sequence (5′–3′) | Product sizes (bp) |
| --- | --- | --- | --- |
| ipaH 01 | Forward | CTTAACTGTACTTAAAGCCAGCGATAACAG | 252 |
|  | Reverse | TCCTGTAGTTCACATGGTAATTCTGGTAAG |  |
| ipaH 02 | Forward | CTGATTACTTTTCAGCATGGGATAAATGGG | 223 |
|  | Reverse | GATGCTGGCAATTCTGGTAATGATATTAGG |  |
| ipaH 03 | Forward | GAATTAGGCCTGACAACATTACCTGAAATC | 282 |
|  | Reverse | CAGTAGTTCTGGTAAAACAGGAAGAGAACA |  |
| ipaH 04 | Forward | CAAATAACCCCTTATCAGATCAAACTCTGC | 271 |
|  | Reverse | GAAAAAGAAGTCCATTGTGTAGAAACCTCC |  |
