# Supplementary material for: Development of a recombinase polymerase amplification assay with lateral flow dipstick (RPA-LFD) for rapid detection of Shigella spp. and enteroinvasive Escherichia coli
Source: PLoS One. 2022 Dec 12;17(12):e0278869. doi: 10.1371/journal.pone.0278869 (PMC9744308; doi:10.1371/journal.pone.0278869)
Supplement: S2 Table — (DOCX) [file pone.0278869.s004.docx]

**S2 Table**

| Primer name | Sequence (5′–3′) |
| --- | --- |
| ipaH 003 | GAATTAGGCCTGACAACATTACCTGAAATC |
|  | Biotin-CAGTAGTTCTGGTAAAACAGGAAGAGAACA |
| ipaH probe11 1111 | FAM-TGAATGCGTCCCACAATCAACTAATCACAC-THF-ACCCACACTCCCCATAT-C3-spacer |
| ipaH probe2 | FAM-TATAAGTAAAAATAATTTAAGCTTAATCT-THF-CCCATTGCCTGCGTCCC-C3-spacer |
