# Supplementary material for: Development of a recombinase polymerase amplification assay with lateral flow dipstick (RPA-LFD) for rapid detection of Shigella spp. and enteroinvasive Escherichia coli
Source: PLoS One. 2022 Dec 12;17(12):e0278869. doi: 10.1371/journal.pone.0278869 (PMC9744308; doi:10.1371/journal.pone.0278869)
Supplement: Attachment — Submitted filename: PONE-D-22-22928_review.docx [file pone.0278869.s005.docx]

PONE-D-22-22928

Development of a recombinase polymerase amplification assay with lateral flow dipstick (RPA-LFD) for rapid detection of the bacterium *Shigella* spp. and enteroinvasive *Escherichia coli*

This study developed a recombinase polymerase-based assay followed by lateral flow dipstick (RPA-LFD) detection of Shigella spp. and the enteroinvasive E. coli. The *ipaH* gene common to both the organisms was used as the target for RPA. This naked eye detection method is rapid and specific to the target organisms.

Major comments:

Although the study successfully developed RPA-LFD method, its utility has not been demonstrated beyond identifying pure cultures of bacteria. Precisely, where this assay can be employed? For clinical specimens, foods or water?. The performance of the assay can vary in different matrices. In this context, although the study has accomplished developing a RPA-LFD method for Shigella and EIEC, it has not demonstrated it applicability to any sample type. Apart from the sample matrix, the DNA extraction method also has bearing on the sensitivity of the assay.

The specificity of the assay can also vary depending on the background microbiota, which again can vary across sample types. It is necessary that the specificity of the assay is established using the natural samples.

The sensitivity of RPA-LFT was determined using the plasmid containing cloned fragment of ipaH gene. Ideally, this should have been done using 10-fold serially diluted bacterial cultures (Shigella and EIEC), followed by DNA extraction.

Minor comments

Title: Consider removing “the bacterium” from the title

L40: “pathogens”.

L45-46: Please provide references to this statement.

L50: Please replace “abundant” with “present”

Table 1: Please expand CICC in the foot note, and the source/origin

L95: Please clarify what did you mean by “NCBI was used to design primers”

L99-100: “After mixing, 2.5 µL of Mg(Ac)_2_ (280mM) was added to start the reaction”.

L109: Please delete “Most of..”

L178-180: Please rephrase this sentence to convey the correct interpretation
