# Supplementary material for: Development of a recombinase polymerase amplification assay with lateral flow dipstick (RPA-LFD) for rapid detection of Shigella spp. and enteroinvasive Escherichia coli
Source: PLoS One. 2022 Dec 12;17(12):e0278869. doi: 10.1371/journal.pone.0278869 (PMC9744308; doi:10.1371/journal.pone.0278869)
Supplement: Attachment — Submitted filename: Response to Reviewers.doc [file pone.0278869.s006.doc]

Reviewer: 1

**Q1**: The optimized assay cannot differentiate *Shigella* spp. and enteroinvasive *Escherichia coli*, and they cross-reacted with each other; Hence the title should be revised accordingly. Also, this can be discused in the discussion section.

**A: The title has been modified according to your suggestions and discussed in the discussion.**

**Q2**: What was the rationale for using the *ipaH* gene?

**A: We focused on foodborne pathogens that can produce toxins in food, and the *ipaH* gene is one of the major genes encoding virulence factors in *Shigella* and enteroinvasive *Escherichia coli*. In addition, Hartman et al. ^[1]^ found that *ipaH* gene has multi-copy nature and exists on both chromosome and invasion plasmid, which makes it superior to other single-copy virulence genes. At the same time, Lampel and Orlandi ^[2]^ reported that storage before testing would lead to the loss of invasive plasmids. Molecular assays designed to detect single-copy genes on invasive plasmids may fail, so molecular assays targeting *ipaH* genes have a greater chance to detect invasive plasmids or chromosomes.**

**Q3**: Line: 81-82: mention the details of the laboratory from where bacterial strains were procured

**A: The manuscript has been revised according to your suggestion.**

**Q4**: Table 1: typhimurium can be written as “Typhimurium” and non-italicize

**A: The manuscript has been revised according to your suggestion.**

**Q5**: Line 101: mention a reference for this “phenol-chloroform method”

**A: The manuscript has been revised according to your suggestion.**

Reviewer: 2
**Q1**: The specificity of the assay can also vary depending on the background microbiota, which again can vary across the sample types. It is necessary that the specificity of the assay is established using the natural samples.

**A: The actual sample testing experiment has been supplemented according to your suggestion.**

**Q2**: The sensitivity of RPA-LFD was determined using the plasmid containing cloned fragment of *ipaH* gene. Ideally, this should have been done using 10-fold serially diluted bacterial cultures (*Shigella* and EIEC), followed by DNA extraction.

**A: Because the extraction method of DNA sample will also affect the sensitivity of detection, we use standard plasmid for 10-fold gradient dilution in order to pursue the accuracy of detection limit. In the actual sample inspection, in order to achieve the universality of detection, use 10-fold of continuously diluted bacterial cultures (*Shigella* and EIEC) according to your recommendations, and then extract DNA.**

**Q3**: Consider removing “the bacterium” from the title

**A:** **The title has been revised according to your suggestions**

**Q4**: L40: “pathogens”.

**A: The manuscript has been revised according to your suggestion.**

**Q5**: L45-46: Please provide references to this statement.

**A: The manuscript has been revised according to your suggestion.**

**Q6**: L50: Please replace “abundant” with “present”

**A: The manuscript has been revised according to your suggestion.**

**Q7**: Table 1: Please expand CICC in the foot note, and the source/origin

**A: The manuscript has been revised according to your suggestion.**

**Q8**: L95: Please clarify what did you mean by “NCBI was used to design primers”

**A: Primers were designed and screened using the primer-BLAST function of the National Center for Biotechnology Information (NCBI).And the manuscript has been revised.**

**Q9**: L99-100: “After mixing, 2.5 µL of Mg(Ac)_2_ (280mM) was added to start the reaction”.

**A: The manuscript has been revised according to your suggestion.**

**Q10**: L109: Please delete “Most of..”

**A: The manuscript has been revised according to your suggestion.**

**Q11**: L178-180: Please rephrase this sentence to convey the correct interpretation

**A: The manuscript has been revised according to your suggestion.**

**Reference**

1. Hartman, A. B., M. Venkatesan, E. V. Oaks, and J. M. Buysee. Sequence and molecular characterization of multicopy invasion plasmid antigen, *ipaH* of *Shigella flexneri*. J. Bacteriol. 1990; 172: 1905–1915.
2. Lampel, K. A., and P. A. Orlandi. Polymerase chain reaction detection of invasive *Shigella* and *Salmonella enterica* in food. Methods Mol. Biol. 2002; 179: 235–244.
