# Supplementary material for: Development of a recombinase polymerase amplification assay with lateral flow dipstick (RPA-LFD) for rapid detection of Shigella spp. and enteroinvasive Escherichia coli
Source: PLoS One. 2022 Dec 12;17(12):e0278869. doi: 10.1371/journal.pone.0278869 (PMC9744308; doi:10.1371/journal.pone.0278869)
Supplement: Attachment — Submitted filename: Response to Reviewers.doc [file pone.0278869.s007.doc]

Dear reviewer:

Thank you very much for your comments. We have revised our manuscript according to your suggestions. And the detail information is following.

Sincerely,

Yuanhong Xie

Reviewer: 2

**Q1**: However, the title might require modification. It says "....... detection of the *ipaH* gene from *Shigella* spp. and enteroinvasive *Escherichia coli*". Here you goal is not just to detect *ipaH* gene. Instead, you are using this gene as a marker to detect two species of bacteria. Therefore, the title can be "Development of a recombinase polymerase amplification assay with lateral flow dipstick (RPA-LFD) for rapid detection of *Shigella* spp. and enteroinvasive *Escherichia coli*

**A: The title has been modified according to your suggestions.**
